# Suppression of Pain in the Late Phase of Chronic Trigeminal Neuropathic Pain Failed to Rescue the Decision-Making Deficits in Rats

**DOI:** 10.3390/ijms22157846

**Published:** 2021-07-22

**Authors:** Suresh Kanna Murugappan, Li Xie, Heung Yan Wong, Zafar Iqbal, Zhuogui Lei, Aruna Surendran Ramkrishnan, Ying Li

**Affiliations:** 1 Department of Neuroscience, College of Veterinary Medicine and Life Sciences, City University of Hong Kong, Hong Kong, China; skmurug@polyu.edu.hk (S.K.M.); lixie7@cityu.edu.hk (L.X.); heunwong5-c@my.cityu.edu.hk (H.Y.W.); ziqbal2-c@my.cityu.edu.hk (Z.I.); zhuogulei2-c@my.cityu.edu.hk (Z.L.); aramkrish3@cityu.edu.hk (A.S.R.); 2 Department of Biomedical Sciences, College of Veterinary Medicine and Life Sciences, City University of Hong Kong, Hong Kong, China; 3 Centre for Regenerative Medicine and Health, Hong Kong Institute of Science and Innovation, Chinese Academy of Sciences, Hong Kong, China; 4 Centre for Biosystems, Neuroscience, and Nanotechnology, City University of Hong Kong, Hong Kong, China

**Keywords:** anterior cingulate cortex, trigeminal neuropathic pain, decision making, early or late phase, tetrodotoxin, synaptic plasticity, theta oscillations, cognitive deficits, chronic constriction injury, riluzole

## Abstract

Trigeminal neuropathic pain (TNP) led to vital cognitive functional deficits such as impaired decision-making abilities in a rat gambling task. Chronic TNP caused hypomyelination in the anterior cingulate cortex (ACC) associated with decreased synchronization between ACC spikes and basal lateral amygdala (BLA) theta oscillations. The aim of this study was to investigate the effect of pain suppression on cognitive impairment in the early or late phases of TNP. Blocking afferent signals with a tetrodotoxin (TTX)-ELVAX implanted immediately following nerve lesion suppressed the allodynia and rescued decision-making deficits. In contrast, the TTX used at a later phase could not suppress the allodynia nor rescue decision-making deficits. Intra-ACC administration of riluzole reduced the ACC neural sensitization but failed to restore ACC-BLA spike-field phase synchrony during the late stages of chronic neuropathic pain. Riluzole suppressed allodynia but failed to rescue the decision-making deficits during the late phase of TNP, suggesting that early pain relief is important for recovering from pain-related cognitive impairments. The functional disturbances in ACC neural circuitry may be relevant causes for the deficits in decision making in the chronic TNP state.

## 1. Introduction 

Infraorbital nerve chronic constriction injury (ION-CCI) is a well-established chronic trigeminal neuropathic pain (TNP) injury animal model that produces prolonged mechanical allodynia and hyperalgesia, and is usually difficult to treat by analgesics and surgical intervention. Cumulative evidence suggests that TNP affects cognitive functions, but little is known about the underlying mechanisms. An enhanced responsiveness of nociceptive neurons in the spinal trigeminal nucleus (SpV) to afferent input (i.e., central sensitization) has been well demonstrated [1,2,3]. In humans, fMRI studies have shown enhanced activation of the SpV, thalamus, and anterior cingulate cortex (ACC) in patients with TNP [4,5], suggesting dysfunction of the brainstem sensory components and higher brain emotional pain system. Recently, we provided direct electrophysiological evidence of synaptic changes in the SpV and higher brain regions in the TNP state [6]. We characterized that long-term enhancement of synaptic transmission occurs in the SpV after trigeminal nerve injury. Afferent hyperactivity is a critical contributor for development of synaptic plasticity within a few hours after nerve injury [7].

Spinal–brainstem–spinal positive feedback loop facilitates the maintenance neuropathic pain states [8], yet pharmacological and surgical manipulation of these centers produced short-term but not long-term relief from allodynia [9], turning attention to other supraspinal areas [8,10]. Ascending multisynaptic pathways from the trigeminal ganglion TG through the SpV and medial thalamus (MT) to the anterior cingulate cortex (ACC) have been identified [11]. Human brain-imaging studies [4,5] have shown enhanced activation in these areas in response to stimuli of the trigger zone in patients with TNP. The anterior cingulate cortex (ACC) is a major cortical component of the limbic loop system, and its functional relationship to emotional and motivational responses, including affective interpretation of pain, has been well described [12,13,14,15,16,17]. Our previous studies have identified the enhanced neuronal responses in the perigenual ACC (pACC) to visceral stimulation in viscerally hypersensitive (VH) rats [18]. ACC activation is critical for pain sensitivity [15,16] and long-term affective memory [17].

Both human and animal studies suggest that chronic pain interferes with cognitive functions [10,19,20]. Orofacial pain-related emotional and cognitive deficits have rarely been studied using experimental models, and little is known about the underlying mechanisms. In the current study, we used a rat gambling task (RGT) [21] to evaluate cognitive function in rats with early or late phases of TNP.

Large-scale neural oscillations are critical for modulating, filtering, and redirecting information in the nervous system. Field potential oscillations can modulate local spike timing, the close coordination of spike timing with the local theta frequency band oscillations predicts the formation of memory in humans [22,23], and they play a major role in the modulation of decision-making behavior in rats [12,13,24]. Ascending multisynaptic pathways from the trigeminal ganglion to the ACC has been identified [11]. Our recent study characterized plasticity in the ACC neural circuitry in the chronic TNP state [6]. By multiple-electrode array recordings in awake rats, we showed that chronic constriction injury (CCI)-induced alterations in the phase locking of ACC spikes to the phase of theta oscillations in the basal lateral amygdala (BLA) in the late phase of TNP, revealing decreased synchronization between ACC spikes and BLA theta oscillations [6].

Afferent hyperactivity is a critical contributor for development of SpV sensitization and synaptic plasticity within a few hours after nerve injury [7]. The causal role of mechanical allodynia in impaired cognitive function in different stages of pain are unclear. We test our hypotheses that block the afferent input in the early phase of TNP is capable to prevent the impaired decision making in rats. On the other hand, impaired inter-area communications in the ACC-BLA network are responsible for impairing decision making in the later stage of TNP.

## 2. Results

### 2.1. Mechanical Nociceptive Behavior

**Figure 1:**
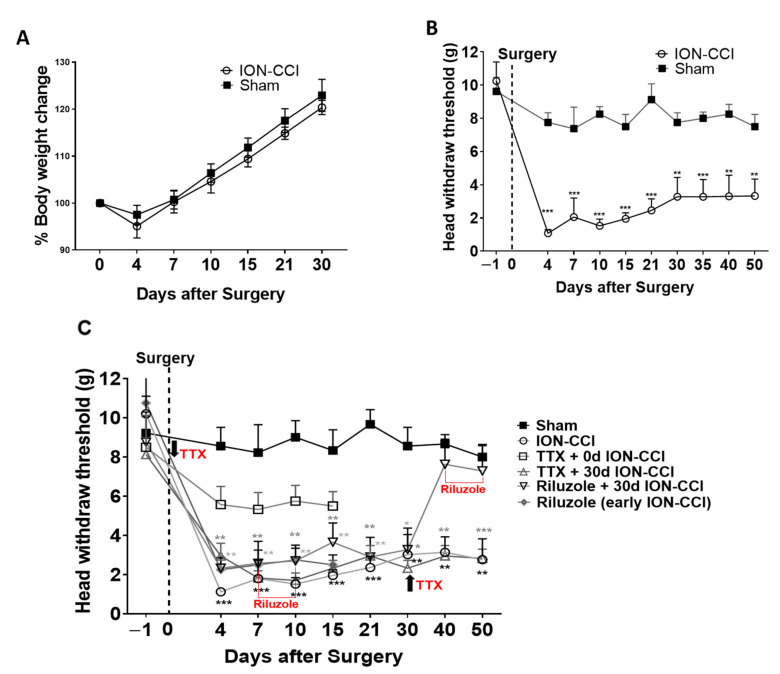
Mechanical nociceptive pain behavior in trigeminal neuropathic pain rats with various pharmacological interference. (**A**) The percentage change of body weight in sham and ION-CCI rats during 30 days following surgerycompared to that of bodyweight on pre-surgery day. Statistical significance was determined by two-tailed unpaired *t*-test. (**B**) Time course of changes in the head-withdrawal threshold to mechanical stimulation of the orofacial skin region ipsilateral to the constricted nerve site in sham rat and ION-CCI rat. Results are presented as mean ± SEM, n = 8 for sham group and n = 8 for ION-CCI group. Statistical significance was determined by two-tailed unpaired *t*-test. ION-CCI vs. sham ** *p* < 0.01, *** *p* < 0.001. (**C**) Drug treatment groups for pain relief are compared with sham and ION-CCI rats. For immediate treatment group, TTX treatment was provided immediately on the day of CCI surgery, whereas for late treatment group, TTX treatment was provided 30 days after CCI surgery in ION-CCI rats. Bilateral intra-ACC infusion of riluzole provided for 5 days in the early stage does not relieve from pain. For late riluzole treatment, intra-ACC riluzole treatment was provided from day 40 repeatedly every 12 h for 4 days, having been shown to relieve the mechanical allodynia in ION-CCI rats on days 40–50. Results are presented as mean ± SEM, n = 10 for TTX immediate after ION-CCI group, n = 8 for TTX after 30-day ION-CCI group, and n = 8 for riluzole after 30-day ION-CCI group. Statistical significance was determined by two-way ANOVA, followed by multiple comparisons adjusted by the Bonferroni’s test, ION-CCI vs. sham ** *p* < 0.01, *** *p* < 0.001; TTX (30 d ION-CCI) vs. sham ** *p* < 0.01, *** *p* < 0.001, riluzole (30 d ION-CCI) vs. sham * *p* < 0.05, ** *p* < 0.01, *** *p* < 0.001.

The body weight of rats for a period of 30 days were noted and percentage of body weight change compared to body weight on day 0 (day of surgery). Both ION-CCI and sham rats showed a decrease in body weight gain on day 4 following surgery, after which their body weight gain increased in a similar pattern for 30 days. There was significant increase in body weight of both ION-CCI and sham rats compared to pre-surgery body weight (F_(time)2.173,30.43_ = 1339, *p* < 0.001; two-way ANOVA), and there were no significant differences in body weight increase rate in ION-CCI rats compared to that of sham rats (F_(surgery)1,14_ = 1.550, *p* = 0.2336; Figure 1A). The mechanical threshold to orofacial stimulation in the sham-operated rats remained in the similar range as the pre-surgery baseline. In contrast, ION-CCI surgery markedly decreased the mechanical pain threshold in the ipsilateral side of the facial region, with the difference in pain threshold first being detected on the fourth day and peaking in the first week. The ION-CCI pain sensitivity was significantly higher than that of sham (F_(9,120),interaction_ = 7.610, *p* < 0.001; F_(6,186),time_ = 9.744, *p* < 0.05; F_(1,14),surgery_ = 33.07, *p* < 0.001, two-way ANOVA repeated measures; Figure 1B) and remained as such at least for 50 days. These results are consistent with previous reports suggesting that ION-CCI surgery in rats evokes pain that is severe in the first week and causes mechanical allodynia for at least 120 days [25].

### 2.2. Effects of Drug Treatment on Pain Perception

TTX Treatment in Early and Late Phase of TNP:

In a subset of rats, TTX was implanted on the ION above the site of constriction during the early stage, immediately after ION-CCI surgery (F_(36,333), interaction_ = 2.586, *p* < 0.001; F_(9,333), main effect time_ = 19.04, *p* < 0.001; F_(4.37), main effect treatment_ = 10.75, *p* < 0.001, two-way ANOVA repeated measures). These animals showed a stable head withdrawal pain threshold of 6–7 g over a period of 15 days, which did not significantly differ from the pre-surgery head withdrawal threshold and sham group (*p* > 0.05, TTX + 0 d ION-CCI vs. sham post hoc Bonferroni test; Figure 1C). These results indicate that local release of TTX during the early stage can minimize the pain transmission from the injured region and provide significant relief from mechanical allodynia. TTX implanted in the late stage, 30 days after ION-CCI surgery, was unable to rescue the animals from mechanical allodynia. The pain sensitivity of the TTX-treated rats did not differ from that of untreated ION-CCI rats but was significantly different from that of sham-operated rats (at all post-surgery days *p* < 0.05, TTX +30 d ION-CCI vs. sham post hoc Bonferroni test; Figure 1C).

### 2.3. Decision-Making Deficit in ION-CCI Rats

**Figure 2:**
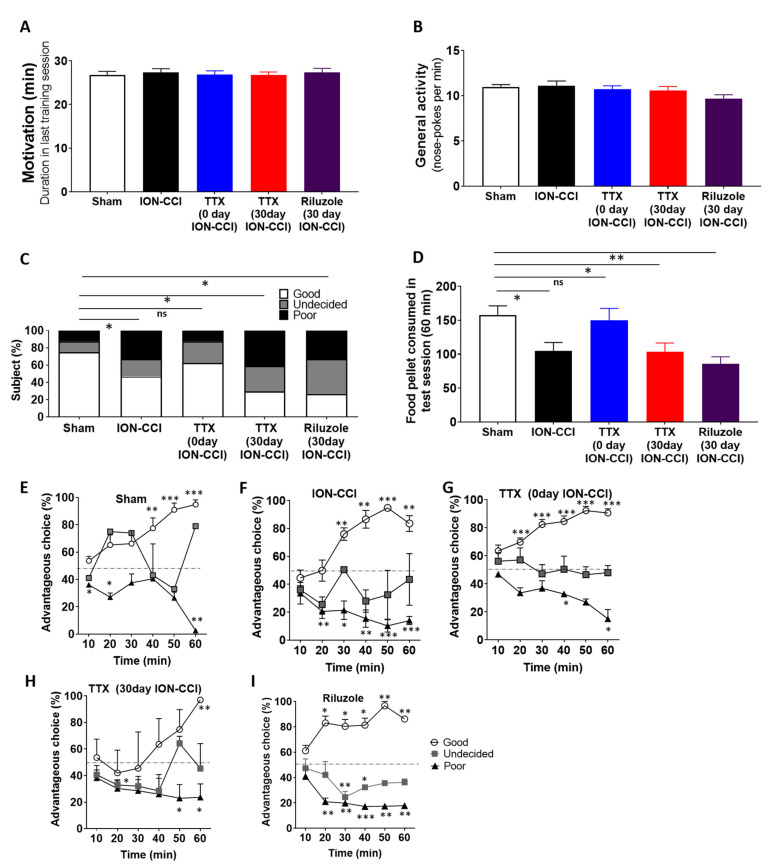
Assessment of decision-making behavior in ION-CCI rats with various pharmacological interference. (**A**) Motivation is measured as the duration taken to consume 100 food pellets during the last T2 training session. Motivation level is maintained at an optimum level for each rat before RGT task. All the rats were equally motivated for the task, and there was no significant difference in the motivation levels between the five groups. (**B**) General activity measured as the total number of nose-pokes made per minute. No significant difference was detected in the general activity between the 5 groups: sham; ION-CCI; and different pharmacological treatments, i.e., immediate TTX treated after ION-CCI, TTX treated 30 days after ION-CCI, and riluzole treated after 30 days ION-CCI. (**C**) The rats in each group were divided into 3 types of decision making on the basis of the choices during the last 20 min of the task. The different proportions of the three types of decision-makers in sham rats (n = 16); ION-CCI rats (n = 15); and ION-CCI rats with different pharmacological treatment, i.e., immediate TTX treated after ION-CCI (n = 17), TTX treated 30 days after ION-CCI (n = 17), and riluzole treated after 30 days ION-CCI (n = 15). Statistical significance between three types of decision-makers in sham and ION-CCI rats was determined by Kruskal–Wallis test, followed by Dunn’s test. (**D**) The food pellets obtained during the test for sham, ION-CCI, and treatment groups: immediate TTX treated after ION-CCI, TTX treated 30 days after ION-CCI, and riluzole treated after 30 days ION-CCI. Results are presented as mean ± SEM. Statistical significance was determined by one-way ANOVA, followed by Bonferroni’s adjusted *t*-test * *p* < 0.05. (**E**–**I**) Time course of choosing advantageous choices among good, undecided, and poor decision-makers during the RGT task observed in sham (**E**), ION-CCI (**F**), TTX-treated immediate ION-CCI (**G**), TTX-treated after 30-day ION-CCI (**H**), and riluzole administered after 30-day ION-CCI rats (**I**). Statistical significance was determined by one-way ANOVA, followed by one-sample *t*-test * *p* < 0.05, ** *p* < 0.01, *** *p* < 0.001 vs. 50% (chance level).

Rat gambling task was performed to assess the decision-making abilities in a complex, conflicting environment. The responses during the last training session showed that there was no significant difference (F_(4,74)_ = 1.769, *p* = 0.144, one-way ANOVA; Figure 2B) in the general activity and motivational level (F_(__4,74)_ = 0.1399, *p* = 0.967; Figure 2A) between the five groups: sham rats (n = 16), ION-CCI rats (n = 15), immediate day 0-TTX-treated ION-CCI rats (n = 17), late day 30-TTX-treated ION-CCI rats (n = 17), and late riluzole-treated ION-CCI rats (n =15). This is consistent with the previous studies showing that neuropathic pain does not affect the general eating and locomotor activity in the cage [6,26]. This suggests that ION-CCI surgery does not affect basal motor function and motivation for collecting food.

Three types of decision-makers were observed in sham and ION-CCI groups: good decision-makers, poor decision-makers, and undecided decision-makers during the RGT task, as defined by their preference for nose-pokes C and D. However, the proportion of good and poor decision-makers was different in each group, as determined by Kruskal–Wallis test (F_(5,80)_ = 10.78, *p* = 0.0291, Kruskal–Wallis test; Figure 2C). ION-CCI rats were significantly different from sham-operated rats (*p* = 0.0403, post hoc Dunn test; Figure 2C). Sham-operated rats had a greater proportion of good decision-makers (75%) and a smaller number of poor decision-makers (12.5%) and undecided decision-makers (12.5%). In ION-CCI rats, the proportion of good decision-makers was reduced (40%) and the number of poor decision-makers was increased (40%) compared to that of sham rats. The food pellet consumed in the last testing session was significantly different among the 5 groups (F_(4,74)_ = 5.561, *p* = 0.0006, one-way ANOVA; Figure 2D). ION-CCI rats collected fewer food pellets during the test session compared to sham rats (*p* = 0.0478, post hoc Bonferroni *t*-test; Figure 2D).

### 2.4. Early TTX Treatment Prevented Decision-Making Impairment in ION-CCI Rats

We performed the RGT in immediate d0-TTX-treated ION-CCI rats (n = 19). Two of the rats showed a preference to a specific nose-poke during the training session as well as on the testing day and were excluded from further analysis. The analysis was performed for the remaining 17 rats. The general activity and motivational level of the TTX drug-treated ION-CCI rats were not significantly different from sham and ION-CCI rats. These immediate TTX-treated ION-CCI rats were not significantly different from sham-operated rats (*p* = 0.7041, post hoc Dunn test; Figure 2C). However, TTX-treated ION-CCI rats were significantly different from untreated ION-CCI rats in the RGT (*p* = 0.0493, post hoc Dunn test; Figure 2C), showing an increase in the proportion of good decision-makers (64.7%) compared to untreated ION-CCI rats (40%), with a marked decrease in poor decision-makers (11.8%) compared to untreated ION-CCI rats (40%). During the course of the RGT task, the proportion of choosing advantageous choices in the good and poor decision-making rats were clearly distinct from the chance level (50%) in all the 5 groups (* *p* < 0.05, ** *p* < 0.01, *** *p* < 0.001, one-sample *t*-test; Figure 2E–I). The food pellets consumed by immediate TTX-treated ION-CCI rats during the test session showed no significant difference compared with that of sham rats (*p* = 0.9918, Bonferroni post hoc; Figure 2D).

The RGT was performed on 17 rats with late TTX treatment implanted 30 days after ION-CCI. In contrast to the early TTX rats, these showed a significant decrease in the proportion of good decision-maker rats (29.4% vs. 75% sham) and increase in the proportion of poor decision-maker rats (41.2% vs. 12.5% sham) compared with sham rats (*p* = 0.0105, Dunn’s test; Figure 2C). There was a non-significant reduction in the total number of the food pellets consumed by the TTX-treated rats (*p* > 0.05, one-way ANOVA; Figure 2D).

### 2.5. Riluzole Treatment in Early and Late Phase of TNP

Repeated bilateral ACC-riluzole infusion every 12 h from day 6 to day 10 after surgery did not attenuate the mechanical allodynia either during or following the end of infusion and showed significantly lower pain thresholds than sham rats but not significantly different from untreated ION-CCI rats (days 7, 10 after ION-CCI surgery, ** *p* < 0.01 vs. sham; *p* > 0.05 (n.s.) vs. ION-CCI; post hoc Bonferroni test; Figure 1C). In contrast, repeated bilateral intra-ACC infusion of riluzole every 12 h from day 40 to day 45 after surgery did reduce the mechanical allodynia for at least 5 days after completing riluzole treatment (days 4–30 ** *p* < 0.01 vs. sham; days 40 and 50, *p* > 0.05 (n.s.) vs. sham; Figure 1C). Riluzole infusion in the late stage provides pain relief, and therefore we studied whether pain relief by riluzole in the late stage rescues decision making.

### 2.6. Riluzole Treatment Did Not Rescue Decision-Making Impairments

The RGT was also performed on 10 ION-CCI rats, starting 35 days after surgery with repeated riluzole administration into the ACC. This treatment showed a significant decrease in the proportion of good decision-makers (33.3% vs. 75%) compared to sham rats, and a significant increase in the proportion of poor decision-makers (33.3% vs. 12.5%) compared to sham rats. The proportions of the 3 response groups in 35-day late riluzole-treated ION-CCI rats were different from sham (*p* = 0.0337, Dunn’s test; Figure 2C). There was a significant reduction in the total number of the food pellets consumed by the later riluzole-treated rats, as shown in Figure 2D (*p* = 0.0028, Bonferroni post hoc test).

### 2.7. Riluzole Suppressed the Neural Activity in ION-CCI Rats

**Figure 3:**
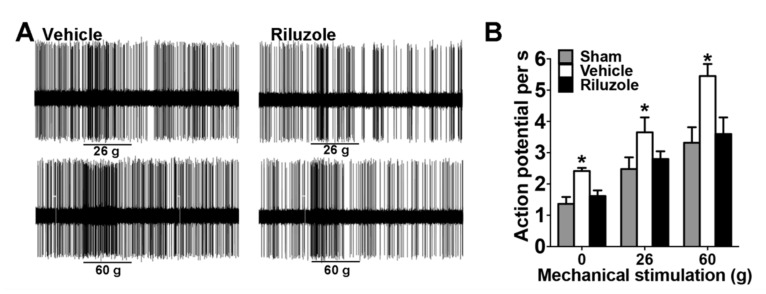
Riluzole suppressed the neural sensitization in ION-CCI rats. (**A**) Representative recordings of ACC mechanical excited neurons in response to graded pressures in anaesthetized CCI rats. (**B**) Local infusion of riluzole into the ACC decreased the neuronal firing rates induced by mechanical stimuli in CCI rats (n = 12 neurons). Statistical significance was determined by two-way ANOVA, followed by multiple comparisons adjusted by the Bonferroni’s test, * *p <* 0.05, ION-CCI (Vehicle) vs. sham.

To clarify whether neurons in the ACC region were involved in mediating hypersensitivity in ION-CCI rats, we recorded neural activity in the ACC while applying mechanical stimulation in the face in anaesthetized rats. In sham rats (n = 5), a total of 32 nociceptively excited neurons in the ACC were recorded. Sample recordings of ACC neuronal responses to graded orofacial mechanical stimuli are shown in Figure 3A. The firing rates gradually increased in response to 15, 26, and 60 g mechanical pressure (F_(2,26)_ = 178.7, *p* < 0.001, two-way ANOVA; Figure 3B). In ION-CCI rats (n = 5), a total of 12 nociceptively excited neurons were recorded. The neuronal firing rates of these neurons also significantly increased in response to gradually increased orofacial mechanical pressures, and the activity at all levels of nociceptive stimulation was increased by at least 50% compared with those of sham rats. Further, to validate if riluzole can suppress the sensitized state in ION-CCI rats, we analyzed the spike field coherence and phase synchrony in awake mice.

### 2.8. Impaired ACC-BLA Spike-Field Phase Synchrony during the Late Stages of Chronic Neuropathic Pain

**Figure 4:**
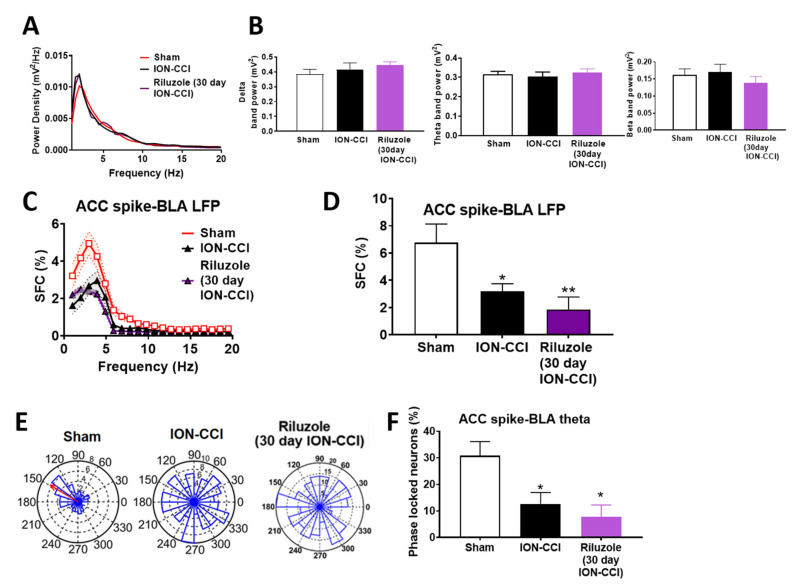
Reduction of spike-field coherence and phase-locking between ACC spikes and BLA theta in ION-CCI rats. (**A**) Power spectral density analysis in the frequency range from 0–20 Hz of the LFP in 30-day ION-CCI, sham-operated rats, and riluzole-treated 30-day ION-CCI rats. (**B**) Power spectral density showing the averaged delta band power (0.5–4 Hz), averaged theta band power (4–10 Hz), and averaged beta band power (10–20 Hz) in the ACC region in ION-CCI and sham-operated rats. (**C**) The SFC distribution of ACC single units as a function of frequencies in sham rats (n = 6), ION-CCI rats (n = 6), and riluzole-treated ION-CCI rats (n = 4). Shadow areas represent SEM. (**D**) Averaged SFC values in theta band show reduction of theta band SFC in ION-CCI rats compared to that in the sham rats. (**E**) Examples show polar-histograms of the ACC spike timing-BLA theta phase distribution of a single unit recorded in the ACC in one sham rat, a single unit in ACC of one ION-CCI rat, and a single unit of one riluzole-treated 30-day ION-CCI rat. The red arrow in the polar-histogram of sham rat represents angle of the mean resultant vector. (**F**) Averaged percentages of phase-locked neurons show a significantly decrease in ACC single-unit spikes phase-locking to BLA theta band field potential in ION-CCI rats and riluzole-treated ION-CCI rats compared to that in sham rats. Results are shown as mean ± SEM (* *p* < 0.05, ** *p* < 0.01 by one-way ANOVA followed by Bonferroni’s adjusted *t* test).

To identify the changes taking place after ION-CCI in cortical regions of awake rats, we compared the single unit recordings from the ACC and the LFP in the BLA region, and also computed the SFC values between the ACC spikes and the BLA LFP. Power spectral analysis was performed during 120 s quiet waking state in sham-operated rats, ION-CCI rats, and riluzole-treated ION-CCI rats. There was no significant difference in the power spectra in the ACC region between the three groups (Figure 4A). The averaged theta, delta, and beta band power did not differ significantly between the three groups (theta band: sham vs. ION-CCI vs. riluzole-treated ION-CCI rats, one-way ANOVA, F_(2,13)_ = 0.2462, *p* = 0.7853; delta band: sham vs. ION-CCI vs. riluzole-treated ION-CCI rats, one-way ANOVA, F_(2,13)_ = 0.6131, *p* = 0.5566; beta band sham vs. ION-CCI vs. riluzole-treated ION-CCI rats, one-way ANOVA, F_(2,13)_ = 0.5970, *p* = 0.5649; Figure 4B).

**Figure 5:**
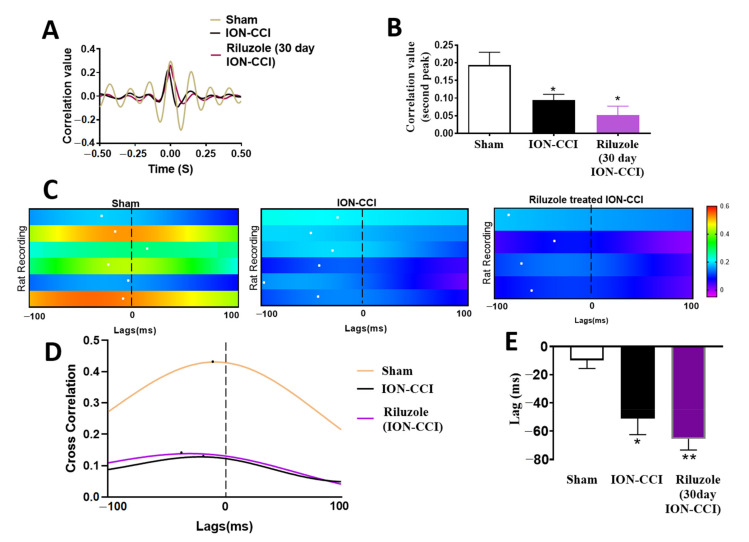
Disrupted BLA-ACC LFP theta coherence and information flow in ION-CCI rats. (**A**) Cross-correlation analysis of theta-filtered LFP recorded in the BLA and ACC revealed a reduction of synchronization in theta band in ION-CCI rats compared to sham rats. (**B**) Averaged cross-correlation value of second positive peak, which represents synchronization of theta band LFP between the BLA and the ACC, showing that the synchronization was decreased in ION-CCI rats. Riluzole treatment in ION-CCI rats also did not rescue synchronization between the BLA and the ACC. (**C**) Lag estimate for sham rats (left; n = 6), ION-CCI rats (centre; n = 6), and ION-CCI rats with riluzole treatment (n = 4) by cross-correlation of instantaneous amplitude of field potential recorded from BLA and ACC. Warmer colors indicate higher cross-correlation peaks or greater phase-locking strength. White dots represent the lags at which the cross-correlation coefficient peaks occurred. (**D**) Representative distributions of cross-correlation coefficient at different lags from examples of one ION-CCI rat (red line) and one sham rat (blue line). Dark stars show the peaks of the cross-correlation. (**E**) The reduction of mean lags at the peak in ION-CCI rats and ION-CCI rats with riluzole treatment were compared to sham rats. * *p* < 0.05, ** *p <* 0.01 by one-way ANOVA followed by Bonferroni adjusted *t*-test.

ION-CCI rats (n = 6) displayed a decrease in SFC in the low-frequency range compared to sham rats (n = 6) (n = 32 neurons for control group, n = 28 neurons for ION-CCI group, and n = 22 neurons for riluzole-treated ION-CCI group, F_(2,1580)_ = 41. 38, *p* < 0.001, two-way ANOVA; Figure 4C). The average SFC values were significantly different in all three groups: sham, ION-CCI, and riluzole-treated ION-CCI (F_(2,79)_ =5.886, *p* = 0.0041, one-way ANOVA). The averaged SFC values in theta range (4–10 Hz) were significantly decreased from 6.78% ± 1.35% to 3.21% ± 0.53 in the ION-CCI rats (*p* = 0.044, Bonferroni post hoc test; Figure 4D). In addition to that, the percentage of phase-locked neurons in the theta phase decreased from 30.83% ± 6.80% in sham rats to 12.66% ± 4.22% in the ION-CCI rats (F_(2,13)_ = 6.253, *p* = 0.0125, one-way ANOVA, *p* = 0.045, Bonferroni post hoc test; Figure 4E,F). We also computed the cross-correlation between the LFP filtered data from ACC and BLA (Figure 5A). The second peak of the cross-correlation coefficient notably decreased in all the ION-CCI rats (F_(2,13)_ = 6.551, *p* = 0.0108, one-way ANOVA, *p* = 0.0493, sham vs. ION-CCI, post hoc Bonferroni test; Figure 5B). Previous references indicate that the ratio of second peak to the positive peak of the cross-correlation coefficient peak was positively related to the electrical activity in the lateral amygdala (LA) and the CA1 area of the hippocampus in freely behaving fear-conditioned mice [27]. These results suggest that reduced BLA to ACC information flow might contribute to undecided and poor decision-making performance. Among the sham rats, four of them showed high cross-correlation between ACC and BLA and two of them showed lower cross-correlation values. BLA-ACC lag estimates showed that the cross-correlation peak was located at negative lags among ION-CCI and sham rats (Figure 5C), signifying an information flow directionality from the BLA to the ACC. Sham group rats depicted a consistent information flow from the BLA to the ACC (mean lag = −9.833 ± 5.730; n = 6 sham rats; Figure 5D,E), whereas the information flow is disrupted in the ION-CCI (mean lag = −51.17 ± 11.30; Wilcoxon signed-rank test, n = 6 ION-CCI rats, *p* = 0.0126 vs. sham; Figure 5D,E).

### 2.9. Riluzole Treatment Did Not Restore the ACC-BLA Spike-Field Phase Synchrony during the Late Stages of Chronic Neuropathic Pain

Riluzole was infused into the ACC region, 20 min before recording the signals from the ACC and BLA region. Riluzole-treated ION-CCI rats (n = 4) displayed a decrease in SFC values in the low frequency range compared to that of sham rats (n = 7) (n = 22 neurons for riluzole-treated ION-CCI group, F_(2,1580)_ = 41.38, *p* < 0.001, two-way ANOVA; Figure 4C). The averaged SFC values in theta range (4–10 Hz) of the riluzole-treated rats were 1.86648% ± 0.5965% significantly lesser than the sham rats compared to the sham group (t = 2.893, *p* = 0.0058, Bonferroni post hoc *t* test; Figure 4D). In addition to that, the percentage of phase-locked neurons in the theta phase was lesser compared to sham rats (9.79% ± 4.255% in the riluzole-treated ION-CCI rats; F_(2,15)_ = 3.775, one-way ANOVA; Figure 4F).

Riluzole administration in ION-CCI rats also showed a decrease in the peak of the cross-correlation coefficient compared to that of sham-operated rats (F_(2,13)_ = 9.447, *p* = 0.02, one-way ANOVA; Figure 5A,B) and similar to that of untreated ION-CCI rats. All the riluzole-treated ION-CCI rats (n = 4) showed low cross-correlation between ACC and BLA (mean lag = −65.50 ± 7.848; Wilcoxon signed-rank test, n = 4 riluzole-treated ION-CCI rats, *p* = 0.0033 vs. sham; Figure 5C–E). These data reveal that riluzole administration does not improve the synchronization within the ACC-BLA circuitry.

### 2.10. Dysfunction of Astrocytic Glutamate Reuptake and Astrocyte Neuron Metabolic Coupling in Chronic Neuropathic Pain

**Figure 6:**
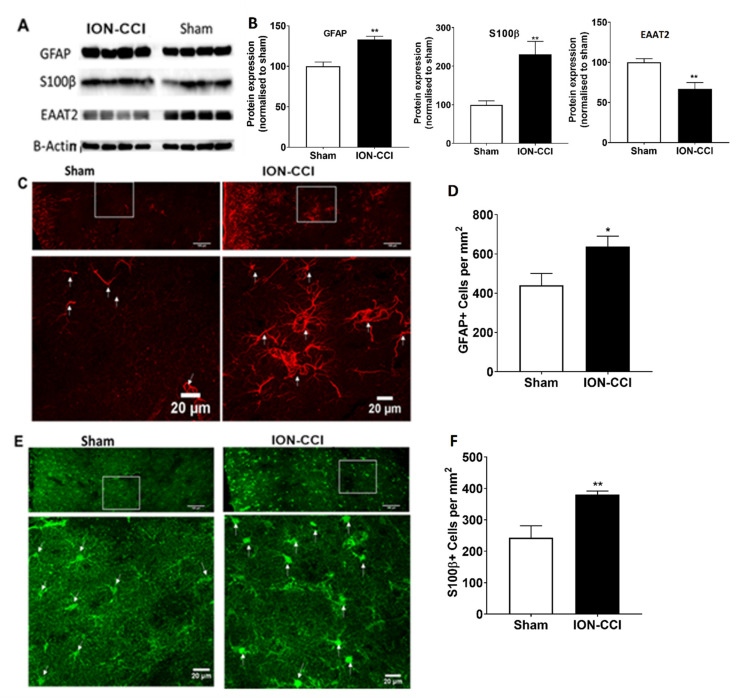
Astrogliosis in the anterior cingulate cortex (ACC) region in ION-CCI and sham-operated rats. (**A**) Brain tissue extracts were collected 30 days after the ION-CCI surgery or sham surgery. Immunoblot images showing GFAP, S100β, and EAAT2 protein bands from the ACC tissue lysate extracts obtained from ION-CCI and sham-operated rats. (**B**) GFAP and S100β protein content were increased in the ION-CCI rats, and glutamate transporter (EAAT2) was decreased in TNP rats, indicating cortical astrogliosis. (**C**) Representative confocal images showing increase in GFAP+ cells in the ACC. The top panel is at lower resolution showing the whole ACC region analyzed, and the bottom panel is at higher resolution showing layer IV. The white arrow marks point the cells in the ACC region. (**D**) The GFAP+ cells were increased by 44% in ION-CCI rats in the ACC region. (**E**) Representative confocal images showing increase in S100β+ cells in the ACC. The top panel is at lower resolution showing the whole ACC region analyzed, and the bottom panel is at higher resolution showing layer IV. The white arrow marks point the cells in the ACC region. (**F**) The S100 β+ cells were increased by 38% in ION-CCI rats in the ACC region. Statistical significance was determined by unpaired t-test, ION-CCI vs. sham, * *p* < 0.05, ** *p <* 0.01.

We then investigated whether the desynchrony between the ACC and BLA is related to the astrocytic dysfunctions that occur under chronic neuropathic pain. We quantified the expression of GFAP and S100β, sensitive and reliable markers of reactive astrocytes that respond to CNS damage and disease [28,29]. Immunohistochemical images of GFAP+ (Figure 6C) and S100β+ cells (Figure 6E) showed more astrocytes in ION-CCI rats. Quantification revealed that the total number of GFAP+ cells in the ACC was significantly increased (by 44.94%) to 638.29 ± 52.61 cells/mm^2^ in ION-CCI rats compared with 440.37 ± 59.9 cells/mm^2^ in controls (unpaired *t*-test, n = 4, t6 = 2.48, *p* = 0.00123; Figure 6D). S100β+ cells in the ACC were also increased (by 56.30%) to 380.91 ± 10.98 cells/mm^2^ in ION-CCI rats compared with 243.69 ± 37.24 cells/mm^2^ in controls (n = 6, t6 = 3.533, *p* = 0.0458; Figure 6F). Further, Western blot analyses of protein extraction in the ACC showed increases of GFAP (n = 4, t6 = 5.551, *p* = 0.0014; Figure 6A,B) and S100β expression (t6 = 3.754, *p* = 0.0095; Figure 6A,B) in ION-CCI rats. Previous studies revealed that astrocyte functions in glutamate–glutamine cycle is mediated by glutamate uptake into astrocytes through the excitatory amino acid transporter 2 (EAAT2) [30,31]. Accordingly, we tested the expression of EAAT2 and found that it was reduced to 65.47% compared with control (t6 = 3.366, *p* = 0.00151; Figure 6A,B; Figures S1–S3) in ION-CCI rats. These observations revealed that a decrease in glutamate uptake may affect the balance of the glutamate–glutamine cycle.

## 3. Materials and Methods

### 3.1. Animals

Adult male Sprague-Dawley rats (300–350 g) were used in this study. The animals were housed in plastic cages in a standard temperature-controlled room (25 °C) with 12 h light/dark cycle and habituated for at least 5 days before any experiment; they were given access to food and water ad libitum, except for the period of the rat gambling task (RGT). A total of 126 animals were used in this study. All experimental procedures were carried out in accordance with the guidelines established by National Institute of Health and approved by the Committee on the Use and Care of Animals at the City University of Hong Kong and the licensing authority for conduction experiments of Department of Health of Hong Kong (ref no. Rev2(18-173) in DH/SHS/8/2/5 Pt.4).

### 3.2. Chronic Trigeminal Neuropathic Pain Model

Chronic constriction of the infraorbital nerve (ION) was performed to develop chronic trigeminal neuropathic pain. The surgical procedures to ligate the ION was adapted from previous publications [6,32]. Briefly, rats were anesthetized with an inhalant anesthetic (mixture of 3–4% isoflurane and pure oxygen for medical usage) delivered through a face mask. The skin above the eye was shaved, and ophthalmic cream was applied to the cornea to protect from drying. A curvilinear incision was made 2 mm above the left eye along the curve of the frontal bone, and the muscle tissue close to the bone was then gently dissected laterally using a scalpel blade until the contents of the orbit could be gently retracted laterally. Once the eye was retracted, the ION was seen lying approximately 8 mm deep within the orbit on the maxillary bone. A total of 5 mm of the ION was gently freed from the surrounding connective tissue with fine jeweler’s forceps, and the two ligatures were then made 4 mm apart around the nerve using 5.0 chromic gut suture. After ligating ION, the incision above the eye was sutured, and the rat was then kept undisturbed to recover. For sham-operated rats, similar surgical procedures were performed, except that constriction of the ION was not performed.

### 3.3. Allodynia and Hypersensitivity Testing

Mechanical pain sensitivity in the facial region was assessed as described in a previous publication [33,34]. Rats were stationed individually into a plastic cage to acclimatize to the testing environment for 2 h. Mechanical sensitivity was determined with a series of 8 Von Frey filaments (VFF) (Semmes-Weinstein monofilaments, Stoelting, Wood Dale, IL, USA) that produced a bending force of 0.6, 1.0, 2.0, 4.0, 6.0, 8.0, 10.0, and 15.0 g. After habituation, stimuli were applied using the VFF in an increasing force order between the whisker pad region and the surgical site on the ipsilateral side of CCI surgery, until a well-defined responsive behavior, such as brisk withdrawal of the head or/and an attack/escape reaction, was triggered [25]. The minimal VFF force to trigger these behaviors for at least 5 times out of 10 stimulations was considered as the head withdrawal. VFF testing to assess mechanical allodynia in the facial region was performed 1 day before and 4, 7, 10, 15, 21, 30, 40, and 50 days after surgery.

### 3.4. Rat Gambling Task (RGT)

The rat gambling task is a behavioral paradigm used to assess the decision-making abilities in rats to choose between immediate gratification and long-term gains (food reward). The RGT task was performed on day 14 after surgery for three experimental groups: sham-operated rats, ION-CCI rats, and ION-CCI rats with immediate TTX treatment. For ION-CCI rats with TTX treatment or riluzole treatment after 30 days ION-CCI surgery, the RGT task was performed on days 35–40. The experimental set-up and procedures for the gambling task have been described in our earlier studies [6,12,21,23,35,36]. During training, each rat was placed in the chamber for 40 min each day in order to gradually learn the association between the nose-poke action and the release of a food pellet (45 mg). The entire training phase usually lasted 6–8 days. During training phase, the rats were free to choose by nose-poking between the 4 apertures (A–D), and each nose-poke choice (A–D) was associated with the delivery of equal number of food pellets. The rats were trained within a series of training sessions until attaining 100 pellets within 30 min. The test procedure lasted 60 min and was performed on the day after training completion. Rats were free to choose between the four apertures (A–D) as they were during the training phase; however, each choice was associated with different food reward and different likelihood of a timeout penalty. Nose-pokes A and B were associated with delivery of 2 food pellets but with a penalty of 222 s and 444 s and a probability of penalty of 50% and 25%, respectively. Moreover, nose-pokes C and D were associated with delivery of 1 food pellets and with a penalty of 6 s and 12 s and a probability of penalty of 50% and 25%, respectively. In the long run, the maximum benefit of collecting food reward associated with C and D was five times than that of A and B. Hence, the nose-pokes C and D were more advantageous for the rats to collect as many food pellets as possible. The good decision-making rats learned the uncertain, risky choices in nose-poke A and B within few trials and therefore avoided choosing those nose-pokes and progressively favored non-risky, advantageous choices in nose-pokes C and D during the latter part of the task, whereas poor decision-making rats chose nose-pokes A and B for immediate gratification, and the rats failed to learn these outcomes chose the options randomly.

The percentage of advantageous choices [(C + D)/(A + B + C + D) × 100%] during the last 20 min and the total food rewards obtained across the test were used to identify the decision-making behavior of the rats. The rats that choose more than 70% of advantageous choices during the last 20 min of the task were classified as good decision-makers, if they chose less than 30% of the advantageous choices then they were classified as poor decision-makers, and those rats that chose between 30% and 70% were classified as undecided.

### 3.5. TTX-Elvax Preparation and Implantation

Elvax polymer has been developed to promote sustained release of the biomolecules and has been found not to affect the general activity of the animals [37]. TTX-Elvax implanted in brain regions (e.g., hippocampus, somatosensory cortex, and cerebellum) has shown to block the neural activity for a period of at least 12 days [33,38], as well as to prevent the increase of dendritic spine formation in the early phase of sciatic nerve ligated neuropathic pain; the effect lasted for more than 6 days [39]. Ethylene vinyl acetate copolymer resin (Elvax) containing tetrodotoxin (TTX) was prepared as described previously [40]. Elvax beads (100 mg) were dissolved in 1 mL dichloromethane (100 mg/mL) and mixed homogenously with 20 μL of DMSO containing 2% Fast Green and 20 μL of 1 mM TTX solution. The Elvax-TTX solution mixture was plated on a Petri dish kept in dry ice, transferred and kept at –70 °C for 1 h, and then placed at –20 °C for overnight to allow the dichloromethane to evaporate. The final concentration of TTX in Elvax was approximately 200 μM. For the implantation of an Elvax piece, the ION site was opened as described above, and the connective tissue surrounding the ION nerve, 8–10 mm from the constricted nerve site, was gently freed using fine jeweler’s forceps for 5 mm. A small piece of Elvax (2 × 4 mm) containing 3.2 nanomoles of TTX was implanted surrounding the ION nerve. The Elvax-TTX cuff released TTX for a period of at least 6 days. Elvax implantation alone (i.e., Elvax-saline) did not cause deficits in general behavior and did not affect pain sensitivity [35]. TTX-Elvax implantation was performed immediately after ION-CCI surgery for the immediate TTX-treated ION-CCI group, and 30 days after ION-CCI for the TTX treated 30-day ION-CCI rat group.

### 3.6. Riluzole Administration

Bilateral cannulas were implanted in the ACC region (stereotaxic coordinates: anterior-posterior (AP) = 3.0 mm, medial–lateral (ML) = ±0.6 mm, dorsal–ventral (DV) = 2.8 mm from dura), and riluzole was infused in freely moving rats. Riluzole (2-amino-6-(trifluoromethoxy)-benzothiazole) was dissolved in 10% cyclodextrin in 0.9% saline. The mixture was bilaterally infused into intra-ACC (20 µg/10 µL) [41] every 12 h for a period of 4 days to study the effects of ACC-riluzole in mechanical allodynia and decision making.

### 3.7. Multiple-Channel Electrophysiological Data Analyses

Electrophysiological recordings were conducted on the rats on days 8–14 after infraorbital nerve ligation. Detailed procedures have been published in our previous publications [6,12,23,24,36,42]. Data analyses were performed using a combination of tools in Matlab, Neuroexplorer, and Offline Spike Sorter.

#### 3.7.1. Power Spectral Analysis

The power spectral density of the LFPs recorded in the ACC region was analyzed. The raw LFPs of ACC were filtered between 1 and 20 Hz using fourth-order Butterworth. The band power was defined as the area under the curve of the corresponding frequencies, and the band power from each animal were averaged over the 16 channels in the ACC.

#### 3.7.2. Spike Sorting

Single-unit spike sorting was performed using Offline Spike Sorter software (Version 4, Plexon Inc., Dallas, TX, USA). A single unit was identified using the criterion of finding <3% of the spikes in the refractory period of 2 ms in the inter-spike interval (ISI) histograms. Detailed procedures have been described in our recent publications [6,12,23,24,36,42].

#### 3.7.3. Spike-Field Coherence

SFC between the spikes recorded in the ACC and the averaged LFP from the BLA were quantified. Only neurons with at least 50 spikes during the period analyzed were used for SFC analysis. For every spike, a segment of the LFP data centered on the spike ± 480 ms long was extracted. The spike triggered average (STA) was calculated as the mean of all these sections. Then the frequency spectrum of the STA (fSTA) was calculated using MATLAB functions for multitaper analysis. The average of these individual frequency spectra resulted in the spike-triggered power as a function of frequency STP(f). Finally, the SFC was calculated as the fSTA over the STP(f) as a percentage. SFC(f) = [fSTA(f)/STP(f)] * 100%.

#### 3.7.4. Phase-Locking of Single Neuron Spikes to the Theta Oscillation

We plotted the phase distribution and analyzed Rayleigh test using custom written MATLAB scripts [43] in order to study the angular distribution of ACC spikes in relation to the ongoing BLA theta oscillation to help clarify the strength of phase-locking. To ensure the validity of the statistical results, we used only neurons with at least 50 spikes during the period analyzed for phase-locking estimation. A neuron was considered phase-locked in the theta range if the *p*-value was below the threshold of 0.0023.

#### 3.7.5. Synchronized Theta Oscillations between ACC and BLA by Cross-Correlation Analyses

The synchronized theta LFP activities between ACC and BLA were evaluated by computing cross-correlograms. Theta-filtered LFPs from the ACC and BLA were aligned, and the LFP in the ACC was chosen as the reference. Pearson correlation coefficients were calculated with a lagging time ranging from −0.5 to 0.5 s with small bins (2 ms). The cross-correlation curves were smoothed with a Gaussian filter. The cross-correlograms from valid electrode channels in the ACC and BLA were analyzed for the second positive peak located around 0.2 s lagging time as a quantitative measure that represents theta activity at about 5 Hz.

### 3.8. Experimental Design and Statistical Analysis

All rats were evenly distributed into a sham-treated negative control group, an ION-CCI-treated positive control group, or a TTX-treated ION-CCI intervention group. These rats were then distributed into follow-up behavioral in order to examine the effects of chronic trigeminal neuropathic pain on sensation and decision making. Data are expressed as mean ± SEM, and statistical significance was analyzed with GraphPad Prism v7.0 (GraphPad, San Diego, CA, USA) or SPSS v19.0 (SPSS, Chicago, IL, USA). Data were analyzed with one-way or two-way factorial ANOVA analysis followed by Bonferroni’s post hoc tests for multiple comparisons where appropriate. The data showing the proportion of decision-makers is presented in ordinal coordinates with a non-normal distribution; therefore, a Mann–Whitney *U* test was performed to assess significance. A value of *p* < 0.05 was considered statistically significant for all comparisons, except for Rayleigh’s test, where *p* < 0.0023 (0.05/22 frequencies tested) was considered significant phase locking.

## 4. Discussion

The patient with trigeminal neuropathic pain (TNP) may present to the dentist with persistent and severe pain, but no clearly identifiable clinical or radiographic abnormalities. The mechanisms involved in the transition of acute pain into chronic pain, such as prolonged “pain memory” known as “atypical odontalgia” [41] and “phantom tooth pain” [44], remain unclear. Neuropathic pain is often associated with depressive behavior, driven by bursts of lateral habenula, something that has been well established [45,46]. However, the association of neuropathic pain and cognitive functions have not been studied before. The infraorbital nerve (ION)-chronic constriction injury (CCI) has become the most popular TNP injury animal model due to the robust induction of chronic allodynia and hyperalgesia [39]. An enhanced responsiveness of nociceptive neurons in the spinal trigeminal nucleus to their normal afferent input has been well demonstrated [2,3]. Recently, we used the ION-CCI model to create stable allodynia lasting at least 7 weeks. Electrophysiological recordings demonstrated that, compared with sham rats, ION-CCI rats showed a long-lasting enhancement of the local field potential (LFP) in the trigeminal ganglia (TG)-SpV caudalis (SpVc) synapses, and this enhancement was reduced by blockade of NMDA receptors [6]. This long-lasting LFP enhancement at the first synapse from the TG to the SpV can play a role in the development of hyperalgesia, but the mechanisms governing chronic pain state remain largely unknown.

Voltage-gated sodium channels (VGSC) mediate neuronal excitability and signaling TTX-sensitive VGSC that plays the predominant role in initiating action potentials in pain-sensing sensory neurons. Immediately after nerve injury, this ectopic discharge propagates to spinal cord and higher brain centers. This initial fast-onset pain is mediated by A-fiber nociceptors whose axons are myelinated [47]. Tetrodotoxin, a potent channel blocker of VGSCs, can block these myelinated A-afferents and thereby reduce the initial fast onset of pain. TTX has been studied in different rodent models including formalin-induced pain, spinal nerve ligation, and sciatic nerve constriction models to alleviate pain [39,48,49]. A previous study reported that afferent hyperactivity is a critical contributor for development of SpV sensitization and synaptic plasticity within a few hours after nerve injury [7]. Here, we suppressed neuronal activity from the injured nerve by applying Elvax-TTX locally to the nerve lesion sites at different time points and showed that immediate TTX treatment at the time of injury completely suppressed the CCI-induced allodynia. These observations are consistent with previous work showing that continuous TTX nerve blockade in the injured region during the early phase blocked the development of prolonged mechanical allodynia [50,51]. TTX-Elvax implanted in brain regions (e.g., hippocampus, somatosensory cortex, and cerebellum) has been shown to block the neural activity for a period of at least 12 days [33,38], as well as to prevent the increase of dendritic spine formation in the early phase of sciatic nerve-ligated neuropathic pain, with the effect lasting for more than 6 days (39). However, the same TTX treatment initiated during the later phase (30 days after nerve injury) failed to prevent long-term tactile allodynia. Thus, it appears that pain development relies on spontaneous ectopic activity by injured sensory axons in the early phase, but maintenance of the allodynia over long times does not depend on ectopic activity from the chronically injured nerve.

Recent studies indicate that glial cells, especially astrocytes, contribute to synapse development, synaptic transmission, and neuronal excitability [52]. CNS damage causes activation of astrocytes by undergoing cellular, molecular, and functional change, a phenomena named reactive astrogliosis. Glial fibrillary acidic protein (GFAP) was the first molecular marker to be strongly associated with reactive astrocytes. The anterior cingulate cortex (ACC) is involved in pain processing. Our results demonstrate upregulation of GFAP and S100β immunoreactivity in the ACC, suggesting development of reactive astrogliosis in the brain ACC area in chronic TNP. Investigators have demonstrated that the trigeminal central sensitization involves glial activation [53]. Glutamate is normally rapidly cleared from the synaptic cleft, primarily by astrocytes that express high-affinity excitatory amino acid transporters (EAATs). Glutamate may be converted to glutamine by the astrocytic enzyme glutamine synthetase (GS). Then, glutamine is released from astroglia and taken up by the presynaptic neuron, and converted back into glutamate [30,54], which plays a major role in central sensitization [31]. In this study, we show significant suppression of EAAT2 expression in ACC, suggesting an alteration of the ACC glutamate–glutamine shuttle; moreover, the decreases of glutamate uptake may trigger an excitatory–inhibitory imbalance, and over-excitability of ACC neurons may modulate the descending endogenous analgesia system, including the periaqueductal gray and the rostral ventral medulla [43], facilitating a pain sensation [16]. This study revealed that during the late phase of TNP, astrogliosis occurs in the ACC region and is associated with decreased expression of EAAT2. Riluzole is a drug that enhances glutamate uptake by EAAT2 and also exerts anti-glutamatergic effects through the inhibition of presynaptic glutamate release and enhancement of glutamate transporter activity. Riluzole has been shown to reduce apoptosis and significantly relieve pain in rats with spinal cord compression or visceral hypersensitivity [55,56,57]. Coderre et al. reported that repeated riluzole administration can relieve mechanical allodynia for at least 8 days in rats with CCI-SN; however, the sites of action have not been clarified [51]. We examined the effects of riluzole injections in the ACC on allodynia in the early (day 6 to 10) and late phases (day 40 to 50) after nerve constriction. Remarkably, we showed that ACC riluzole does not impede basal nociceptive responses and allodynia in the early phase but is effective in relieving allodynia during the late phase. These observations suggest that the later phases of allodynia depend on cortical long-term neural plasticity that depends on appropriate glutamate concentrations at ACC synapses. Thus, we propose the existence of two distinct phases of allodynia, the first independent of, and the second dependent on ACC plasticity. Higher brain center facilitation may be required for maintaining of neuropathic pain in the later phase. This identification would allow specific brain targeting and design of medication for chronic orofacial pain disorders.

Human and animal studies suggest that chronic pain interferes with cognitive function [19]. Reward-based learning and decision-making involves active contributions of the orbitofrontal cortex, the ventromedial prefrontal cortex, and the anterior cingulate cortex [58,59]. A number of studies have suggested that changes in the cingulate cortex, the orbitofrontal cortex, and the insula can be looked at as a common “signature” of chronic pain [60,61,62,63]. Decision making under complex and uncertain conditions is a basic cognitive process that needs adaption. In this study, we used a rat gambling task (RGT) [12,13,21,35,36] to evaluate cognitive function after chronic ION-CCI and observed significant decreases of the proportion of good decision-makers from 75% in the control to 46% in the chronic ION-CCI rats. We also found marked increases in the numbers of animals that did not learn the task after chronic ION-CCI. These data provide the first evidence that ION-CCI impairs decision making in rats. We further showed that TTX applied locally to the nerve lesion sites immediately following ION-CCI surgery suppressed the CCI-induced allodynia and also improved decision making. On the other hand, TTX applied in the later phase, after full development of allodynia, failed to prevent decision-making deficits in CCI rats.

Next, we asked whether allodynia plays a key role in causing impairment of decision making, which remains unclear. When riluzole was infused into the ACC during the early stage of TNP, it did not rescue the development of allodynia. However, riluzole infusion in the late stage of TNP significantly attenuated the allodynia but failed to rescue decision-making deficits in rats with TNP. These results indicate that development of allodynia is dependent on peripheral pain signals in the early stage of TNP. ACC is necessary for modulating pain in the late stage of TNP, and the mechanism for decision making is distinct from that pain modulation. Riluzole infusion in the ACC interferes only in the pain modulation.

Brain oscillations can synchronize neurons and create coherent cell assemblies [64]. The BLA and the ACC form an interconnected neural circuit that mediate effort-based decision-making processes [65]. Our earlier electrophysiological study showed a reduction of long-term potentiation in the basolateral amygdala (BLA)-ACC synapses in rats with chronic visceral pain [62]. We showed that phase-locking and synchronization within the ACC and between the ACC and BLA play a major role in the modulation of decision-making behavior in rats. Much evidence suggests that theta rhythms are involved in facilitating the transfer of information between brain regions. In line with this observation, our work here showed that the synchronization of spikes in the ACC to the BLA LFP is decreased after ION-CCI, and this reduced LFP synchronization between the BLA and the ACC was also associated with reduced phase-locking of ACC spikes to the theta oscillations in the BLA. The cross-area spike–LFP–phase-locking in the late phase of TNP suggested that reciprocal connections between the BLA and the ACC are critical for information transfer in this situation. Further, riluzole administration decreased the neural sensitization of ACC neurons but failed to rescue the desynchrony in the BLA-ACC pathway. Thus, the information flow between ACC and BLA remained affected and therefore failed to rescue the decision-making abilities.

## 5. Summary

These studies collectively show that decision-making deficits are associated with pain sensation in the early phase of TNP; however, in the late phase, it becomes independent to the pain sensation per se. In the later phase, chronic TNP affects higher brain region BLA-ACC circuitry. Hence, early pain relief treatment is important for recovering from pain-related cognitive impairments.

## Data Availability

The data presented in this study are available on request from the corresponding author.

## Supplementary Materials

The following are available online at https://www.mdpi.com/article/10.3390/ijms22157846/s1.
