# Supplementary material for: Suppression of Pain in the Late Phase of Chronic Trigeminal Neuropathic Pain Failed to Rescue the Decision-Making Deficits in Rats
Source: Int J Mol Sci. 2021 Jul 22;22(15):7846. doi: 10.3390/ijms22157846 (PMC8346079; doi:10.3390/ijms22157846)
Supplement: Supplementary file 1 [file ijms-22-07846-s001.zip › ijms-1308760-supplementary.pdf]

Supplementary Materials

Suppression of pain in the late phase of chronic trigeminal neuropathic pain failed to rescue the decision-making deficits in rats

Suresh Kanna Murugappan, Xie Li, Heung Yan Wong, Zafar Iqbal, Zhuogui Lei, Aruna Surendran Ramkrishnan, and Ying Li*

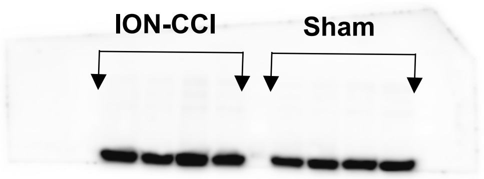

Figure S1. Unprocessed Western blot images for Figure 6A - GFAP.

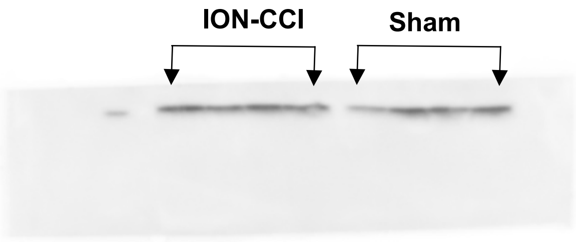

Figure S2. Unprocessed Western blot images for Figure 6A – S100β.

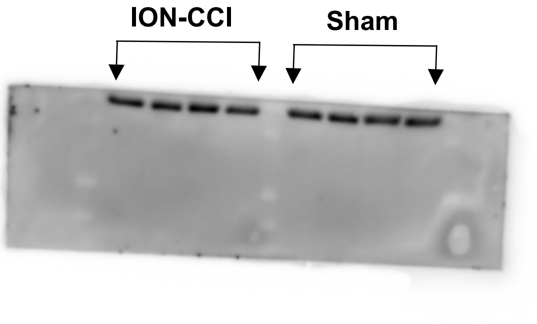

Figure S3. Unprocessed Western blot images for Figure 6A – β Actin.
